# A natural human IgM that binds to gangliosides is therapeutic in murine models of amyotrophic lateral sclerosis

**DOI:** 10.1242/dmm.020727

**Published:** 2015-08-01

**Authors:** Xiaohua Xu, Aleksandar Denic, Luke R. Jordan, Nathan J. Wittenberg, Arthur E. Warrington, Bharath Wootla, Louisa M. Papke, Laurie J. Zoecklein, Daehan Yoo, Jonah Shaver, Sang-Hyun Oh, Larry R. Pease, Moses Rodriguez

**Affiliations:** 1 Department of Neurology, Mayo Clinic, Rochester, MN 55905, USA; 2 Department of Electrical and Computer Engineering, University of Minnesota, Minneapolis, MN 55455, USA; 3 Department of Biomedical Engineering, University of Minnesota, Minneapolis, MN 55455, USA; 4 Department of Immunology, Mayo Clinic, Rochester, MN 55905, USA

**Keywords:** Amyotrophic lateral sclerosis, ALS, SOD1, Gangliosides, GT1b, GD1a, Neurite extension, Axon growth, Neuroprotection, Surface plasmon resonance, SPR, Supported lipid bilayers

## Abstract

Amyotrophic lateral sclerosis (ALS) is a devastating, fatal neurological disease that primarily affects spinal cord anterior horn cells and their axons for which there is no treatment. Here we report the use of a recombinant natural human IgM that binds to the surface of neurons and supports neurite extension, rHIgM12, as a therapeutic strategy in murine models of human ALS. A single 200 µg intraperitoneal dose of rHIgM12 increases survival in two independent genetic-based mutant *SOD1* mouse strains (SOD1G86R and SOD1G93A) by 8 and 10 days, delays the onset of neurological deficits by 16 days, delays the onset of weight loss by 5 days, and preserves spinal cord axons and anterior horn neurons. Immuno-overlay of thin layer chromatography and surface plasmon resonance show that rHIgM12 binds with high affinity to the complex gangliosides GD1a and GT1b. Addition of rHIgM12 to neurons in culture increases α-tubulin tyrosination levels, suggesting an alteration of microtubule dynamics. We previously reported that a single peripheral dose of rHIgM12 preserved neurological function in a murine model of demyelination with axon loss. Because rHIgM12 improves three different models of neurological disease, we propose that the IgM might act late in the cascade of neuronal stress and/or death by a broad mechanism.

## Abstract (precis)

**Summary:** A single peripheral dose of a recombinant natural human IgM increases lifespan and delays neurological deficits in mouse models of human ALS.

## INTRODUCTION

ALS is characterized by the degeneration of anterior horn spinal cord motor neurons and corticospinal tract neurons, resulting in progressive muscle weakness, loss of muscle mass and inevitably death due to respiratory failure. Despite extensive research, the etiology of this disorder is largely unknown, and there are no effective treatments. Only one approved drug, riluzole, minimally improves survival of ALS patients (Stewart et al., 2001). The vast majority of individuals with ALS develop the disease sporadically. However, genes implicated in a minority of individuals with inherited ALS provide mechanistic clues to this disorder (Gurney et al., 1998). Many familial ALS cohorts carry mutations in the superoxide dismutase-1 (*SOD1*), *TDP-43* (*TARDBP*) or fused in sarcoma (*FUS*) genes; however, the number of genes associated with ALS continues to grow, and is now at least 32, including *C9orf72* and *EphA4*, indicating the extent of heterogeneity of the disease (Sreedharan and Brown, 2013). Regardless of whether a patient carries a known ALS-associated gene mutation or is sporadic without a known mutation, disease progression is similar clinically (McGoldrick et al., 2013), suggesting that the underlying motor neuron dysfunction is related. Therefore, reagents efficacious in familial ALS might also aid the far more prevalent sporadic forms.

The generation of transgenic mice carrying human ALS-associated genes with pathological features of ALS has advanced our understanding of the role that these gene products play in disease (Turner and Talbot, 2008). These transgenic mice develop progressive motor neuron loss and neurological deficits and, although not perfectly predictive of human ALS, are useful to screen potential drugs for clinical trial. With the growing list of genes linked to ALS, a candidate reagent cannot be realistically tested in all possible transgenic animal models. However, drugs that act at a final common pathway to protect neurons across several models might lead to a viable human therapy. The most widely used murine models of ALS for preclinical evaluation carry mutated versions of the *SOD1* gene (Scott et al., 2008). The Cu/Zn SOD (copper/zinc superoxide dismutase) enzyme catalyzes the conversion of free superoxide radicals to oxygen and hydrogen peroxide. Mutations in the SOD1 enzyme are associated with familial ALS (Morrison and Morrison, 1999). There seems to be a gain of toxic function of SOD1 (Bruijn et al., 2004), due to conformational instability of the proteins, which interferes with fast axonal transport (Cluskey and Ramsden, 2001). In human ALS, degenerating motor neurons contain abnormal accumulations of insoluble and misshaped protein of both wild-type and mutant SOD1 (Bosco et al., 2010).

Our laboratory has identified natural human monoclonal antibodies (mAbs) that bind to the surface of central nervous system (CNS) cells and have screened them as therapeutics for neurological disorders (Rodriguez et al., 2009). To date, all of our therapeutic human antibodies are of the IgM isotype. Methods were developed to clone, sequence (Ciric et al., 2001) and express large quantities of recombinant IgM required for human studies (Mitsunaga et al., 2002). A recombinant human IgM, rHIgM22, that binds to myelin and promotes remyelination (Warrington et al., 2000) recently completed Phase I clinical trial in patients with multiple sclerosis, without any safety issues. Several related human IgMs that bind to the surface of neurons support robust neurite outgrowth (Warrington et al., 2004; Xu et al., 2011b). Based on evidence that a recombinant form of one of these neurite-promoting human mAbs, rHIgM12, preserved neurological deficits in a virus-induced mouse model of spinal cord demyelination with secondary neuronal degeneration (Wright et al., 2009; Denic et al., 2011), this mAb was tested for its effect on lifespan and axon protection in the SOD1G86R and SOD1G93A mouse models of ALS. We also characterized the antigens and binding affinity of rHIgM12.

**TRANSLATIONAL IMPACT**

**Clinical issue**

Amyotrophic lateral sclerosis (ALS) is characterized by degeneration of anterior horn spinal cord motor neurons and corticospinal tract neurons, resulting in progressive muscle weakness, loss of muscle mass and inevitably death. Despite extensive research, the etiology of this disorder is largely unknown, and there are no effective treatments. Only one approved drug, riluzole, minimally improves survival of individuals with ALS. Thus, there is a critical need to develop neuron-protective drugs. Natural human monoclonal antibodies (mAbs) of the IgM isotype that bind to the surface of central nervous system (CNS) cells are showing promise as novel therapeutics for neurological disorders.

**Results**

In this study, the authors tested the effects of a neuron-binding natural human monoclonal IgM, rHIgM12, on two superoxide dismutase-1 (*SOD1*)-mutant mouse strains, two well-known models of ALS. In these animals, a single intraperitoneal dose of rHIgM12 prolongs survival and preserves spinal cord axons and anterior horn neurons. A small amount of this large human IgM crosses the blood-brain barrier and can be detected in the brain and spinal cord. Thin-layer chromatography and surface plasmon resonance showed that rHIgM12 binds to GD1a and GT1b gangliosides (which are a major type of CNS glycosphingolipids). Both of these gangliosides are crucial components of membrane microdomains. In neuronal cultures, addition of rHIgM12 increases α-tubulin tyrosination levels, suggesting an alteration of microtubule dynamics.

**Implications and future directions**

This study shows that rHIgM12 attenuates degeneration of neurons and axons, possibly by interacting with cell-membrane components and by stabilizing microtubule domains. Previous data showed that rHIgM12 is efficacious in a virus-induced model of multiple sclerosis. The fact that this IgM is therapeutic in two different models of neurodegenerative disease implies a broad ability to protect axons. rHIgM12 is a fully human monoclonal antibody that was cloned from an IgM isolated from an individual who carried it at high levels for many years without adverse effects. This class of natural auto-reactive mAbs, common in the human immunoglobulin repertoire, might be harnessed to enhance the physiological reparative process of damaged neurons and axons.

## RESULTS

### A single peripheral dose of rHIgM12 increases survival of *SOD1* transgenic mice

Because a single dose of the human IgM rHIgM12 preserved neurological deficits in a virus-mediated model of spinal cord demyelination and the IgM bound to the surface of every neuronal type in cultures tested to date, we hypothesized that rHIgM12 might be protective across neuronal disease injury models. We tested whether rHIgM12 could preserve neurons under stress in mouse models of the extreme human neurodegenerative disease, ALS. Efficacy of rHIgM12 was evaluated in two different strains of ALS model mice, SOD1G86R and SOD1G93A. The SOD1G86R mouse strain carries a mutant murine *SOD1* gene and presents with a rapid disease progression and death by 120 days. The SOD1G93A strain carries multiple (up to 23) copies of a mutant human *SOD1* allele (Tu et al., 1996) and presents with a slower disease progression, but ultimately death occurs by 140 days. Weight loss in each model begins at 90-100 days of age, which typically precedes progressive neurodegeneration (Morrison et al., 1998).

**Fig. 1.**
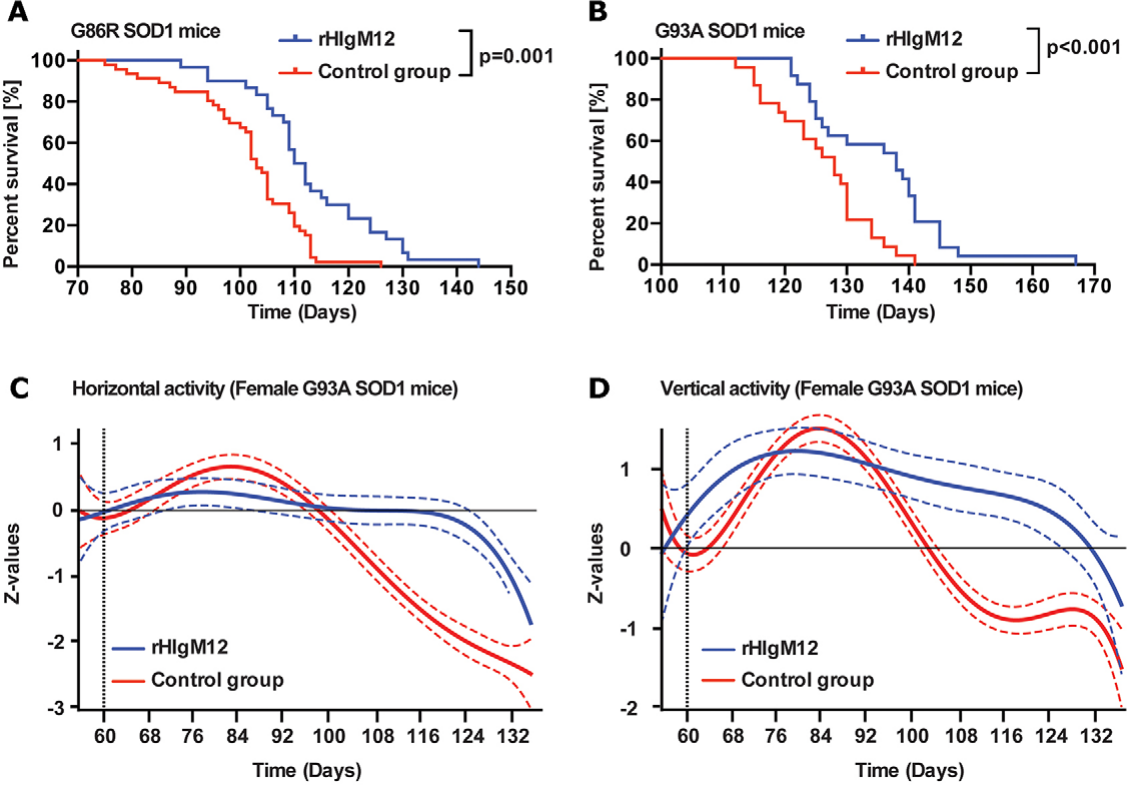
**rHIgM12 enhances survival of SOD1 mice and slows deficit progression.** (A,B) A single 200 μg intraperitoneal dose of rHIgM12 given at 8 weeks of age increased survival of both the SOD1G86R (rHIgM12 *n*=30 females, control *n*=35 females) and SOD1G93A (rHIgM12 *n*=24, control IgM *n*=23) strains of mice. Lifespan was plotted using Kaplan–Meier curves and analyzed using GraphPad Prism (*P*<0.0001, Log-Rank test). (C,D) A subset of female SOD1G93A mice at 56 days of age were placed as groups of five in AccuScan locomotor activity-monitoring boxes and monitored continuously. Baseline activity (beam breaks/hour) collected over 4 days is represented as the horizontal line at 0. Mice were treated with a single dose of 200 μg of rHIgM12 or PBS at 60 days of age. Nocturnal horizontal activity (C) and vertical activity (D) are graphed using a fifth-order polynomial fitting of standardized *z*-values. rHIgM12 treatment distinctly preserved spontaneous movement (C) and rearing (D). The point-wise 95% confidence bands for the polynomial curves are indicated by hashed lines surrounding the red and blue lines and indicate the times of significant differences between groups. Horizontal axis indicates age of mice in days.

A single 200 µg intraperitoneal dose of rHIgM12 or a control human IgM was administered to SOD1G86R mice at 8 weeks of age, well before the onset of neurological deficits. Only a single dose of human antibody was given to prevent any potential immune response elicited by multiple doses of a foreign protein. Treated mice were followed until moribund. Thirty female SOD1G86R mice received rHIgM12, and 35 female mice received a control human IgM. Comparing survival between the two groups revealed that rHIgM12-treated SOD1G86R mice lived significantly longer. Mean survival of the rHIgM12-treated group was 113±12.1 days, whereas mean survival of the control group was 101.7±10.7 days (median difference 8 days, *P*=0.001, Fig. 1A). The first control-group death occurred at 75 days, vs 89 days in the rHIgM12-treated group.

To determine whether this lifespan extension could be observed in a second ALS model, groups of 24 SOD1G93A mice of mixed gender were treated with a 200 µg intraperitoneal dose of rHIgM12 or saline. Comparing survival between the two groups revealed that rHIgM12-treated SOD1G93A mice also lived longer than their control group (mean survival 135.4±11.1 days vs 126±8 days, median difference 10 days, *P<*0.001, Fig. 1B). The first death in the control SOD1G93A mice occurred at 112 days, vs 121 days in the rHIgM12-treated group.

To determine whether neurological deficit progression was also slowed in an ALS model by treatment with rHIgM12, spontaneous horizontal and vertical movements of five female SOD1G93A mice from each treatment group were measured in activity boxes over 80 days as a readout of quality of life, i.e. time in a deficit-free state (Fig. 1C,D). Curve deflection from the 0-horizontal axis indicates a group's change from baseline (activity measured during the 4 days prior to treatment). We found that spontaneous activity of the rHIgM12-treated mice was preserved compared to controls. Horizontal and vertical movements remained at or above their baseline for 16 and 28 days longer, respectively, in rHIgM12-treated mice compared to controls. Performance in both groups eventually shifted below baseline with disease progression. In the same groups of mice, the onset of weight loss was delayed by 5 days for rHIgM12-treated mice compared with controls.

**Fig. 2.**
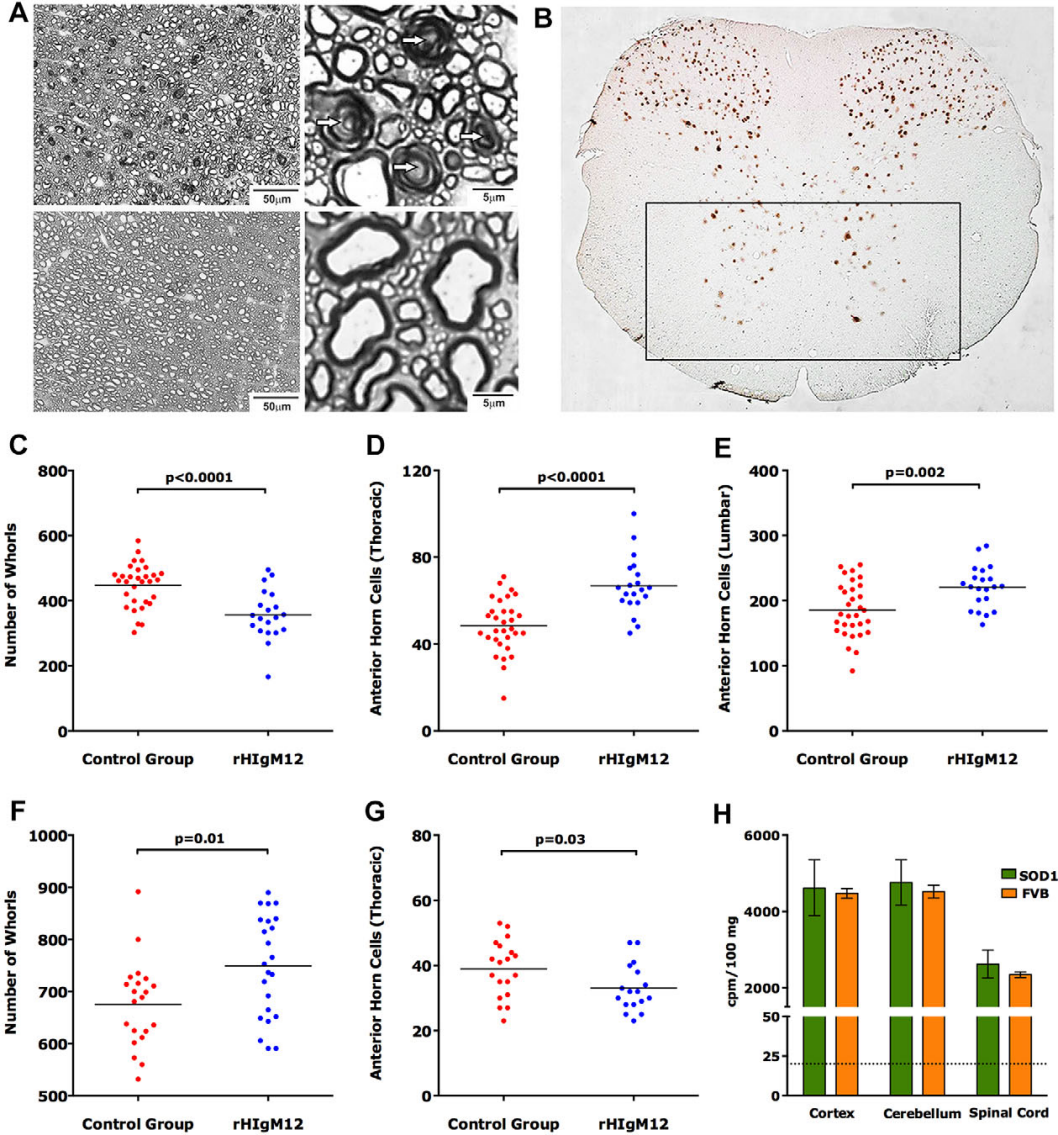
**A single 200 µg dose of rHIgM12 preserves spinal cord neurons in SOD1G86R mice.** Spinal cord axonal injury and motor neuron loss was examined histologically. Myelin whorls, characteristic of degenerating axons, were counted in six sampled areas from a mid-thoracic spinal cord section. (A) Representative mid-thoracic-level micrographs from a SOD1G86R mouse containing numerous myelin whorls (upper panels; arrows) compared to micrographs from an age-matched normal littermate control (lower panels). An analysis of the number of myelin whorls indicated fewer degenerating axons in rHIgM12-treated SOD1G86R mice compared to controls (C, *P*<0.0001, *t*-test). However, this was not true of rHIgM12-treated SOD1G93A mice (F, *P*=0.01). (B) Because human ALS primarily affects the health of motor neurons, anterior horn cells of the spinal cord (outlined in the black rectangle) were quantified across treatment groups. Two mid-thoracic- and two lumbar-level spinal-cord sections were stained with anti-NeuN, which nicely defines spinal-cord neuronal cell bodies. Anterior horn NeuN+ cells were counted in these four cross-sections. A larger number of NeuN+ cells were found at both the thoracic (D) and lumbar (E) levels in spinal cords of rHIgM12-treated SOD1G86R mice compared to controls (*P*<0.0001 and *P*=0.002, respectively, *t*-test). Again, this observation was not confirmed in thoracic-level sections from SOD1G93A mice (G). Each circle represents one animal. All tissue staining, imaging and analyses were performed blinded. (H) A single 50 µg intraperitoneal dose of rHIgM12 (10 million cpm of ^35^S) was administered to 90-day-old female SOD1G86R and FVB mice. 20 h later, cpm/100 µg of brain and spinal-cord tissue was determined. Bars are the mean±s.e.m. of three mice. Background counts for comparable weights of CNS tissue without rHIgM12 were 17-20 cpm (black dashed line).

Spinal cords from all mutant *SOD1* mice, collected at the time of sacrifice for pathological analysis, contained myelin whorls within the white-matter tracts, indicating collapsed myelin initiated by axonal degeneration (Fig. 2A). Even though all spinal cords were collected from mice deemed moribund, rHIgM12-treated SOD1G86R mice contained significantly fewer myelin whorls (Fig. 2C, *P*<0.0001) than controls. In addition, spinal cords of rHIgM12-treated SOD1G86R mice contained more NeuN+ anterior horn neurons (Fig. 2B) at both the thoracic and lumbar levels (Fig. 2D,E), suggesting attenuated neurodegeneration. These two readouts of axon degeneration and neuron preservation were not observed in SOD1G93A mice treated with rHIgM12 (Fig. 2F,G). In fact, there were more myelin whorls and fewer NeuN+ anterior horn neurons in the rHIgM12-treated group. This could be because rHIgM12-treated SOD1G93A mice live longer than controls, accumulating additional spinal cord injury by the time they are scored as moribund.

IgG and IgM subclass immunoglobulins are readily detected in the CNS at low concentrations (Reiber and Peter, 2001; Gold et al., 2012). However, because there is some dispute that large molecules such as antibodies cross the blood-brain barrier (BBB), and reach the CNS when delivered peripherally, we measured the amount of a single dose of isotopically labeled rHIgM12 that reached the brain and spinal cord. ^35^S-labeled rHIgM12 was generated, 50 µg [10 million counts per minute (cpm)] of the IgM was administered intraperitoneally to groups of three 90-day-old SOD1G86R and age-matched normal FVB mice, and tissue was harvested 20 h later. A 200-fold increase in disintegrations per minute (dpm) over background was detected in cortex and cerebellum, and 100-fold increase in dpm over background was detected in the spinal cord. The amount of radioactivity measured in the brain was 0.2%, and in spinal cord 0.03%, of the total dose of isotope administered. The same fraction of the total delivered dose of IgM was detected in the CNS of both SOD1G86R transgenic mice and normal mice (Fig. 2H).

### rHIgM12 binds to the gangliosides GD1a and GT1b

**Fig. 3.**
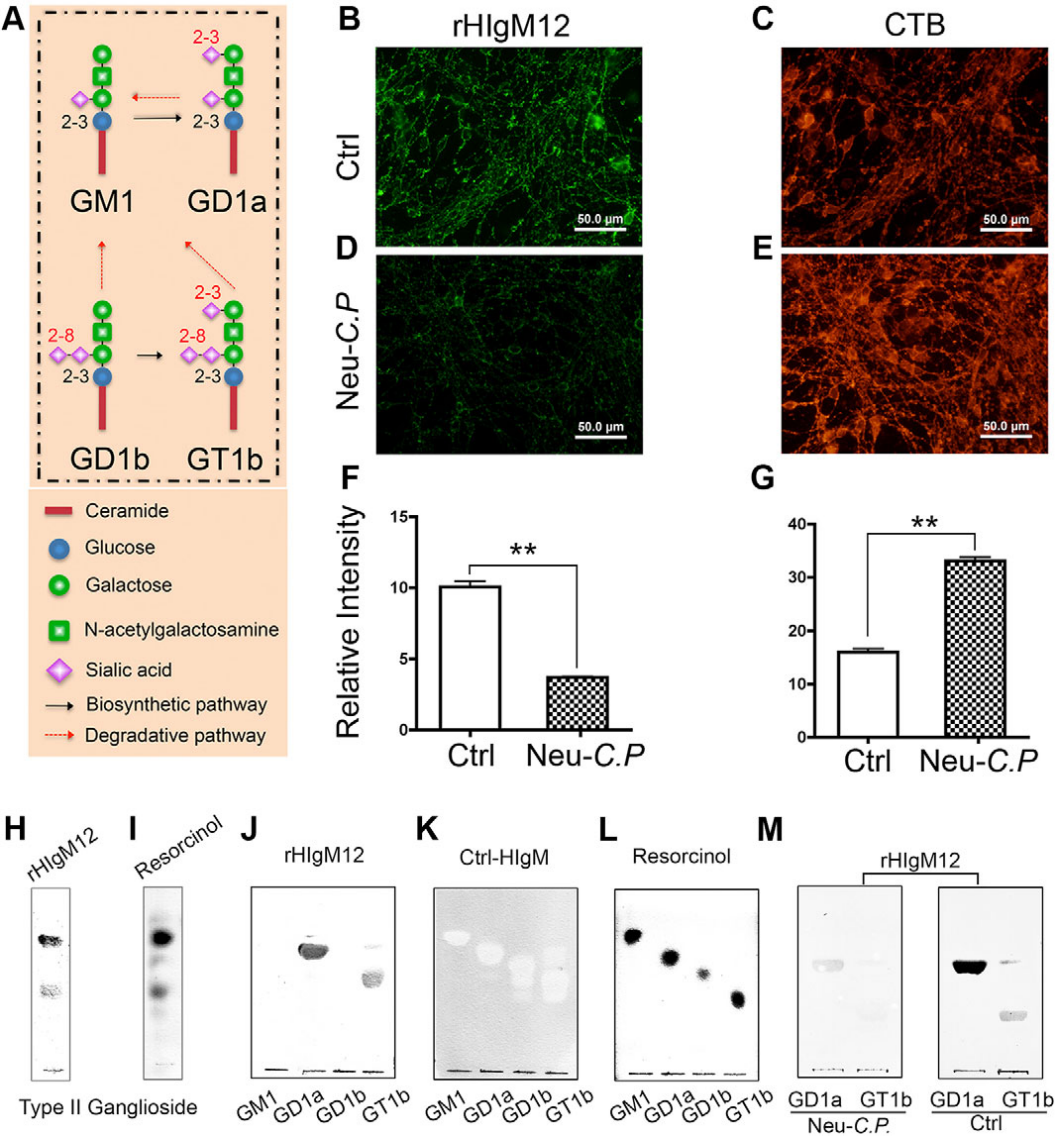
**rHIgM12 binds to carbohydrate epitopes of the gangliosides GD1a and GT1b.** Because rHIgM12 was identified in part by its strong binding to the surface of neurons, which are rich in gangliosides, we tested whether rHIgM12 could recognize prominent brain gangliosides by chromatography. (A) The three major brain gangliosides, GD1b, GT1b and GD1a, are converted to GM1 by Neu-*C.P.*, which hydrolyzes sialic acid at the α2-8 and terminal α2-3 linkages, whereas internal α2-3 linkages remain unaffected. (B-E) Untreated (Ctrl) cerebellar granule neurons in culture (B,C) and those treated with Neu-*C.P.* (D,E) were immuno-labeled with rHIgM12 (B,D, green) and cholera toxin B (CTB) (C,E, red), which specifically binds GM1. Scale bars: 50 µm. (F,G) Quantified surface fluorescent intensities of green (rHIgM12, F) and red (CTB, G) indicated that Neu-*C.P.* treatment decreases rHIgM12 binding by 60%, and increases the binding of CTB twofold (**P<0.01; *t*-test two-sided). Bars are the mean±s.e.m. of three trials. Purified gangliosides from bovine brain were spotted and separated on silica-based high-performance thin-layer chromatography (TLC) plates and then overlaid with rHIgM12 (H). Two bands were detected by rHIgM12 (H), which were also stained by resorcinol (I). Purified GM1, GD1a, GD1b and GT1b were then run on the same TLC plate and incubated with rHIgM12, a control human IgM or stained with resorcinol. rHIgM12 recognized GD1a and GT1b (J), whereas the control human IgM (K) bound to none. Areas of excluded binding observed after extended incubation with the control IgM indicate the position of gangliosides, confirmed in a resorcinol-stained plate (L). Neu-*C.P.* treatment of TLC plates spotted with GD1a and GT1b abolished rHIgM12 binding to both gangliosides (M).

Because rHIgM12 binds strongly to many types of CNS neurons (Warrington et al., 2004; Xu et al., 2011b) we hypothesized that rHIgM12 drives neurite extension through a common membrane component. We previously showed that binding of the serum-derived human IgM, sHIgM12, was sensitive to neuraminidase, which cleaves sialic acids (Warrington et al., 2004). Gangliosides, the major sialic-acid-bearing glycosphingolipids enriched in CNS, are synthesized by adding sialic-acid residues to the core molecule. Neuraminidase from *Clostridium perfringens* (Neu-*C.P.*) hydrolyzes sialic acid α2-8 and terminal α2-3 linkages without affecting the internal α2-3 linkages, converting GD1b, GT1b and GD1a to GM1 (Fig. 3A) (Iwamori et al., 1997). To investigate whether gangliosides are a cell-surface antigen for rHIgM12, granule cell neurons were treated with Neu-*C.P.* and then stained with rHIgM12 and cholera toxin B (CTB), which specifically binds to ganglioside GM1 (Gill and King, 1975). Sialic-acid hydrolysis decreased membrane-bound rHIgM12 (Fig. 3D), whereas CTB binding increased (Fig. 3E), indicating that rHIgM12 binds to complex gangliosides other than GM1. When the overall fluorescent intensity change was measured, Neu-*C.P.* treatment induced a 60% reduction in rHIgM12 binding (Fig. 3F) and a 60% increase in CTB binding (Fig. 3G), suggesting that the rHIgM12 epitopes were converted to GM1.

To determine which complex gangliosides interacted with rHIgM12, bovine-brain gangliosides were separated via high-performance, thin-layer chromatography (HPTLC) overlaid with rHIgM12. rHIgM12 bound to two bands (Fig. 3H), corresponding to the major gangliosides from bovine-brain stained by resorcinol (Fig. 3I). To further characterize these bands, purified bovine-brain GM1, GD1a, GD1b and GT1b were separated on the same HPLC plate and overlaid with rHIgM12. We found that rHIgM12 bound to GD1a and GT1b, but not to GM1 or GD1b (Fig. 3J). A serum-derived human control IgM did not bind to any gangliosides (Fig. 3K). After an extended staining period, ghost bands indicated the location of each ganglioside band, which was also confirmed by resorcinol staining (Fig. 3L). To confirm the rHIgM12 binding specificity, GD1a and GT1b were resolved by HPTLC, treated with Neu-*C.P.* and then overlaid with rHIgM12. Hydrolysis by Neu-*C.P.* eliminated rHIgM12 binding to both GD1a and GT1b (Fig. 3M). This result suggested that α2-3-linked sialic acids attached at an external galactose is the binding site of rHIgM12 to GD1a and GT1b.

### rHIgM12 binding affinity to gangliosides measured by SPR

**Fig. 4.**
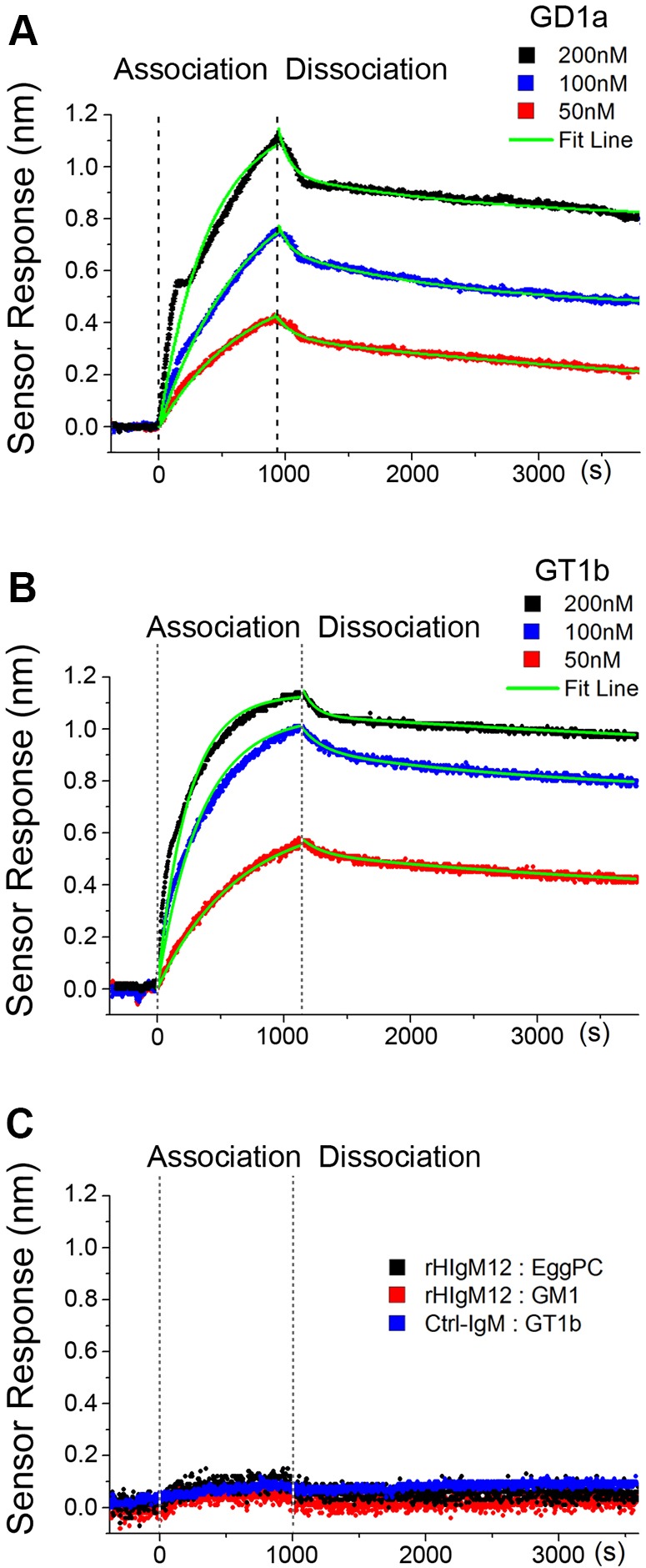
**rHIgM12 binds with high affinity to GD1a and GT1b presented in supported lipid bilayers.** Surface plasmon resonance was used to determine the association/dissociation curves of rHIgM12 binding to 5 mol% GD1a (A) and GT1b (B) in a lipid bilayer. The *K*_D,apparent_ was 42.3±20.6 nM and 24.8±7.9 nM, respectively, for GD1a and GT1b (mean±s.e.m., *n*=4 and 6, collected over 2 and 3 days). (C) Control curves indicated that 100 nM rHIgM12 did not bind to lipid bilayers consisting of only egg PC, or to 5 mol% GM1 presented in a lipid bilayer. In addition, 100 nM of a control human IgM did not bind to GT1b presented in lipid bilayers.

Natural auto antibodies have been traditionally regarded as multivalent molecules with low affinity. Contrary to this notion, our previous experimental evidence indicated that some IgMs actually showed high affinity and avidity to their antigens (Wittenberg et al., 2012), which might explain why those IgMs induced neuroprotection when a small amount crossed the BBB. The high affinity of these IgMs might be due to avidity, which is the accumulated strength of multiple simultaneous binding interactions. These pentavalent IgMs theoretically could bind up to ten receptors, which could increase affinity by 400 times (Crothers and Metzger, 1972). To determine the binding constants and validate the specificity of rHIgM12 to gangliosides presented in a lipid-bilayer environment, nanohole surface plasmon resonance (SPR) was used (Brolo et al., 2004; Dahlin et al., 2005; Im et al., 2010; Wittenberg et al., 2012). Supported lipid bilayers (SLBs) composed of egg phosphatidylcholine (egg PC), with and without GD1a, GT1b and GM1, were formed on silver-film nanohole-array SPR chips (supplementary material Fig. S1A-C). Fluorescence recovery after photobleaching (Axelrod et al., 1976) verified the continuity and two-dimensional fluidity of SLBs, giving diffusion coefficients (Jönsson et al., 2008) for egg PC, GT1b and GM1 membranes of *D*=1.77±0.09, 1.85±0.01 and 2.02±0.03 μm^2^/s (mean±s.e.m.), respectively (supplementary material Fig. S2A,B). rHIgM12 bound to GD1a and GT1b presented in SLBs. To account for multivalent binding between IgMs and multiple gangliosides, SPR-binding curves (derived from spectral data in supplementary material Fig. S3A-E) were fit to a biphasic exponential model (Cooper and Williams, 1999) to extract the association- and dissociation-rate constants. The apparent dissociation constant (*K_D,apparent_*), calculated by the ratio of the dissociation-to-association-rate constants for rHIgM12, was 42.3±20.6 nM to GD1a (Fig. 4A) and 24.8±7.9 nM to GT1b (Fig. 4B) (mean±s.e.m., *n*=4 and 6, collected over 2 and 3 days, respectively). This is strong binding for an IgM antibody. Typical *K_D_* values for IgM-autoantibody binding range from 10^−8^ to 10^−5^ M (Lutz et al., 2009). rHIgM12 did not bind to egg-PC-only SLBs nor to GM1-containing SLBs and a control human IgM did not bind to GT1b-containing SLBs (Fig. 4C). This high-antigen avidity/specificity corresponds to our earlier study of remyelination-promoting mouse IgMs (Wittenberg et al., 2012) and suggests that strong binding might be an overarching feature of IgMs with therapeutic potential.

### rHIgM12 regulates microtubule stability, promoting neurite outgrowth

**Fig. 5.**
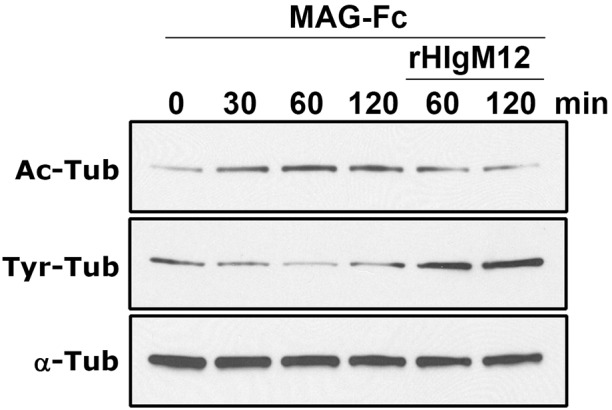
**Soluble MAG-Fc-driven reduction of tyrosinated tubulin is reversed by the addition of rHIgM12.** MAG-Fc binds to GD1a and GT1b on neurons in culture and promotes microtubule stability, including increasing the level of acetylated α-tubulin (Ac-Tub), while decreasing levels of tyrosinated α-tubulin (Tyr-Tub) (Nguyen et al., 2009; Xu et al., 2011b). To test whether rHIgM12 could antagonize the effects of MAG-Fc, cortical neurons in culture were treated with 10 µg/ml of MAG-Fc alone for 2 h. Neuronal lysates probed with antibodies specific for Ac-Tub and Tyr-Tub show that, whereas the total level of α-tubulin remained constant, Ac-Tub increased and Tyr-Tub fell. In comparison, when 10 µg/ml of rHIgM12 was added to the culture media along with MAG-Fc, the levels of Tyr-Tub were dramatically higher 1 and 2 h later and Ac-Tub decreased to baseline 0 time-point levels, indicating active microtubule assembly. The panel shows one of three iterations.

We previously showed that serum-derived sHIgM12 over-rode the inhibitory effects of myelin on neurite outgrowth (Warrington et al., 2004). Myelin-associated glycoprotein (MAG), a major myelin-derived inhibitor of regeneration after neural injury, also binds to GD1a and GT1b (Vyas et al., 2002; Schnaar and Lopez, 2009). MAG binding to neurons results in stabilization of the axon, but inhibits further neurite extension (Pan et al., 2005). Because we previously showed that rHIgM12, added to cortical neurons, substantially increased α-tubulin tyrosination (Xu et al., 2011b), and similar studies showed that MAG decreased α-tubulin tyrosination while increasing acetylation (Nguyen et al., 2009), we tested the two molecules together (Fig. 5). We found that MAG that was fused to the human fragment-crystallizing region (MAG-Fc) and was added to cortical neurons in culture increased tubulin acetylation twofold over 2 h, whereas tubulin tyrosination remained stable (Nguyen et al., 2009; Xu et al., 2011a,b). When rHIgM12 was then added to neuronal cultures in the presence of MAG, tubulin acetylation decreased sevenfold and tubulin tyrosination increased twofold over a period of 2 h. This experiment shows that rHIgM12 can override MAG-mediated tubulin stability, potentially promoting a more dynamic cytoskeleton and neurite extension.

## DISCUSSION

We describe a novel therapeutic approach to protect neurons and potentially extend lifespan across diseases that stress and/or injure neurons as the primary the pathological event. The neurite-promoting human IgM, rHIgM12, which binds to gangliosides GT1b and GD1a, was isolated and cloned from a human patient with monoclonal gammopathy without neurological disease. A recombinant form of this human IgM was tested in two distinct models of neurological disease with very different mechanisms of cell death: (1) a model of axonal injury mediated by a persistent picornovirus and immune attack and, (2) models of ALS driven by a mutation of a protein that is normally involved in preventing oxidative stress. The fact that this IgM is therapeutic in two disparate models (viral and genetic) implies that neuronal protection occurs by a broad mechanism possibly through an indirect mechanism involving immune cells that amplify the single dose of IgM.

A single 200 µg intraperitoneal dose of rHIgM12, given before the onset of disease, increased lifespan in both SOD1G86R and SOD1G93A mice. A single dose of rHIgM12 is sufficient to increase lifespan in the ALS models; however, a single dose might not be the optimum treatment. Treatment with multiple doses of rHIgM12 in animal models is hampered because of a strong anti-human antibody response with subsequent doses that likely inactivates rHIgM12 in circulation. We purposely designed a human antibody for treating patients that could be used without an immune response. We measured fewer degenerating spinal cord axons and more NeuN+ anterior horn neurons in rHIgM12-treated SOD1G86R mice than controls. However, these positive histological readouts were not measured in SOD1G93A mice treated with rHIgM12. Our protocol called for blinded assessment of clinical deficits and all mice were killed at the point at which they were judged to be moribund. SOD1G93A mice treated with rHIgM12 lived 25-30 days longer than SOD1G86R mice treated with rHIgM12. This extended lifespan might allow further accumulation of axon and neuron degeneration. A future goal is to analyze spinal cord pathology of all mice at a point in disease when there is a clear difference in neurological deficits between treated groups, such as at 110 days of age.

In any therapeutic strategy that prolongs survival, there is a need to show that the increase in life, often years in a chronic disease, is associated with an improved quality of life. To assess a potential preservation of neurological deficits, five SOD1G93A mice from each treatment group were randomly selected, housed together and monitored continuously over 80 days in activity boxes. Nocturnal activity is a sensitive measure of spontaneous rodent neurological function. Mice are nocturnal and present with maximal spontaneous performance change at night in the dark. We have published that spontaneous nocturnal horizontal activity of mice provides a strong accurate measure of overall mobility, whereas nocturnal vertical activity provides a measure of rearing (Denic et al., 2011). This same publication describes a preservation of deficits in the Theiler's murine encephalomyelitis virus (TMEV)-induced model of demyelination after treatment with rHIgM12. For both horizontal and vertical activity, the curve for rHIgM12-treated SOD1 mice was often above the control curve. Our analysis indicated that rHIgM12 treatment delays deficit progression in SOD1G93A mice by 16 days; however, a single dose of the IgM does not halt disease.

Of importance, rHIgM12 can be found in the brain and spinal cord after peripheral administration, indicating that this large molecule can cross the BBB. It has been general dogma that IgMs with molecular weights of close to 1,000,000 kDa are too large to cross the BBB. However, there is accumulating evidence that certain antibodies, IgGs and IgMs, can cross the BBB following intraperitoneal or intravenous administration and modify disease (Banks et al., 1991, 2002, 2007; Kozlowski et al., 1992; Pirko et al., 2004). When delivered intraperitoneally, mouse IgMs directed against oligodendrocytes that promote remyelination (Hunter et al., 1997) enter the brain and spinal cord of mice with virus-induced demyelination, and equally the CNS of normal uninfected mice. A human monoclonal IgM that promotes *in vivo* remyelination, rHIgM22, was tracked in mice with CNS demyelination using paramagnetic beads and a 7-Tesla MRI magnet (Pirko et al., 2004). rHIgM22 entered the brain after intraperitoneal injection and accumulated at demyelinated lesions, whereas a control human IgM did not. Human monoclonal IgMs directed against β-amyloid that improved cognitive scores in mice with Alzheimer's-like disease can be found in the brain after peripheral delivery (Banks et al., 2002, 2007), and human monoclonal IgGs directed against the CNS-neuron-specific protein LINGO-1, which improves both the experimental autoimmune encephalomyelitis (EAE) and lysolecithin models of demyelination, are found within the brain and spinal cord of normal rats and rats with encephalomyelitis following intravenous administration (Pepinsky et al., 2011). In all these studies the antibody treatment was therapeutic even though only 0.1% to 0.5% of the total administered dose entered the CNS. It should be stressed that, for many drugs in wide clinical use, very little needs to reach the CNS to produce the therapeutic effect. Only 0.02% of a peripheral dose of morphine or the anti-depressant Fluoxetine enters the brain, yet this level is sufficient for clinical effects (Advokat and Gulati, 1991).

**Fig. 6.**
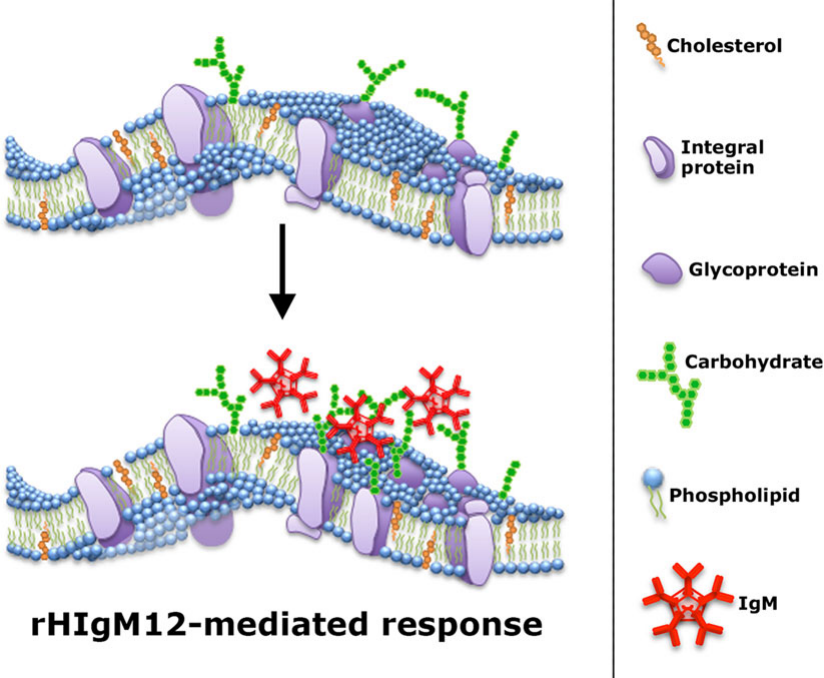
**Working model of a potential rHIgM12 mechanism of action.** Complex gangliosides, including GD1a and GT1b, are present in the plasma membrane anchored by their ceramide tails. The external carbohydrate moieties protrude into the extracellular space and can function as receptors for ligands, including rHIgM12. Each pentameric IgM contains ten potential binding sites. rHIgM12 binding to multiple sites on the plasma membrane could cluster ganglioside-rich membrane microdomains into larger complexes. The clustering of microdomain-associated molecules to a critical level triggers changes on the cytoplasmic face of the membrane, signaling the cell to respond.

Based on our cell-culture studies, we propose a model to explain the tubulin modifications and neurite outgrowth in the presence of rHIgM12 (Fig. 6). rHIgM12 binds to gangliosides on the neuronal surface, rearranges plasma-membrane microdomains and clusters signaling molecules, resulting in a shift of microtubule stability and dynamics (Palestini et al., 2000; Loberto et al., 2003; Xu et al., 2011a,b). This model does not directly explain how rHIgM12 extends survival and delays neurodegeneration *in vivo*.

We used two different binding assays, HPLC with immunoblotting and SPR with supported lipid bilayers, to conclude that rHIgM12 recognizes the gangliosides GT1b and GD1a. This finding is consistent with our previously reported data that rHIgM12 binds to the neuronal membrane, colocalizes with GM1 and cholesterol, and segregates into the lipid-raft fraction and overrides myelin- and MAG-driven inhibition of neurite extension (Warrington et al., 2004; Xu et al., 2011b). Because it contains multiple binding sites, a single rHIgM12 molecule is capable of binding multiple gangliosides simultaneously. To account for the multivalency of rHIgM12, we fit the SPR data to a biphasic exponential binding model. This type of analysis has been previously applied to SPR analysis of IgM binding (Cooper and Williams, 1999) as well as analysis of pentavalent cholera-toxin binding to a number of different gangliosides (Kuziemko et al., 1996). From the analysis, we calculated an apparent equilibrium dissociation constant, *K_D,apparent_*, which captures both affinity and avidity. The molecular avidity of rHIgM12 is implicit in the calculation of the dissociation rate constant because all rHIgM12-ganglioside interactions must dissociate for a molecule to leave the SPR sensor surface. Thus, *K_D,apparent_* takes into account the avidity of rHIgM12 because it is a ratio of dissociation-to-association-rate constants.

Both GD1a and GT1b are enriched components of membrane domains in neurons (Cantù et al., 2011; Sonnino et al., 2013). GD1a and GT1b function as ligands of MAG (Vyas et al., 2002), facilitating key interactions between oligodendrocytes and axons that maintain long-term axonal stability (Sheikh et al., 1999). Both gangliosides are involved in mediating inputs that alter the activation state of the Rho-GTPase signaling network, a key aspect of neurite extension and retraction control. MAG-induced inhibition of axon extension involves the rearrangement of membrane domains (Vinson et al., 2003), including an increased recruitment of p75NTR to the domains (Fujitani et al., 2005). rHIgM12-mediated neuron protection might use the same membrane platforms as therapeutic antibodies directed against Nogo-A and Lingo-1.

To our knowledge, this is the first human natural IgM to extend lifespan in mice with a neurodegenerative disease. The 10-day increase in lifespan in SOD1G93A mice after a single dose of rHIgM12 is comparable with a number of far more cumbersome and lengthy published treatment regimens (Gros-Louis et al., 2010; Lincecum et al., 2010; Jagust, 2013; Koval et al., 2013). Many of these treatment strategies involve intensive intraventricular delivery or continuous treatment over months. The similar lifespan extension suggests that a threshold of repair exists in the SOD1G93A strain that limits improvement of survival. Therefore, many therapeutics will appear equally effective in the primary disease model. Decisions for drug translation to trial in humans should then be driven by cost, ease of delivery and associated risk.

All of our mouse-based studies were run as randomized ‘blinded’ trials using Good Laboratory Practice (GLP)-grade rHIgM12, of sufficient quality for human trial. Only a single dose of human IgM was administered to each mouse because it is a foreign protein; however, if tested in humans with ALS, treatment would likely involve multiple or continuous dosing of rHIgM12. We have performed significant dosing safety studies of rHIgM12 in normal mice and in mice with inflammatory and chronic neurological disease. Virtually no toxicity was identified in complete tissue pathology or blood chemistry analysis. rHIgM12 is derived from a true human IgM identified from a patient with Waldenstrom's macroglobulinemia (Rodriguez et al., 2009) that has carried high levels of the monoclonal protein for years without detriment. This suggests that rHIgM12 will be safe. Natural antibodies are an emerging class of therapeutics that might offer benefit across multiple diseases.

## MATERIALS AND METHODS

### Recombinant human IgM

rHIgM12 was expressed in CHO cells (Xu et al., 2011b). The predominant IgM expressed in the serum of a Waldenstrom's macroglobulinemia patient was sequenced. Plasmids expressing heavy- and light-chain coding sequences for the IgM were transfected into target cells. Cell lines were selected with increasing doses of methotrexate, sub-cloned and expanded to a stable clone producing the desired IgM, which was quantitated by ELISA and tested for potency. Recombinant IgM was purified from culture supernatant using a three-column high-performance liquid chromatography (HPLC) sequence to greater than 97% purity in a GLP-certified commercial facility. The final packaged IgM was validated using titrated potency assays of binding to the surface of neurons in culture and support of neurite extension. rHIgM12 can be obtained for non-commercial experimental use by contacting the corresponding authors. A material transfer agreement between the requestor's institution and Mayo Clinic will be required. The control human IgM used was isolated from human serum. It was characterized (Warrington et al., 2004) and found to not bind to the surface of neurons in culture or live tissue slices, nor support neurite extension in cell culture. Informed consent was obtained from all patients for the use of serum samples for research purposes.

### Transgenic mice, histology and morphological analysis

All animal protocols adhered to National Institutes of Health (NIH) guidelines and were approved by the Mayo Foundation Institutional Animal Care and Use Committee (IACUC). FVB-Tg(SOD1*G86R)M1Jwg/j mice (Ripps et al., 1995) (referred to as SOD1G86R in the manuscript) were obtained from Jackson Labs (stock number 005110; Bar Harbor, ME). The colony was maintained in house by crossing Tg(SOD1*G86R)M1Jwg/J males harboring the transgene with wild-type FVB females. Animals were housed under a 12-h light/12-h dark cycle with free access to food and water. For genotyping, tail biopsies of 21-day-old pups were subjected to a standardized PCR protocol (Jackson Labs) after a restriction-enzyme digest. Probe sequences were: SOD1-forward, 5′-GACATCATTGTTCATCC-3′; SOD1-reverse, 5′-AATGATGGAATGCACTCCTGA-3′. B6SJL-Tg(SOD1*G93A)1Gur/J (referred to as SOD1G93A in the manuscript), also from Jackson Labs (Stock #2726), were genotyped by Jackson Labs and provided by Prize4Life (http://www.prize4life.org/page/als).

At 60 days of age, mice received a single 200 μg dose of rHIgM12 or control human IgM in a volume of 500 μl saline via intraperitoneal injection. A minimal sample size of 24 mice per treatment group was used according to European ALS/MND group recommendations (Ludolph et al., 2010) and those of the ALS Therapy Development Institute (Scott et al., 2008). Mice were weighed weekly and sacrificed for pathology when unable to right themselves within 30 s. All mice included in the study carried *SOD1* transgenes per pre-established genotype criteria. One male SOD1G93A mouse was excluded owing to death from non-neurological disease. All experiments were performed in a blinded manner. Mice were randomized for treatment by one investigator, treated using coded samples, weighed and scored for neurological deficits, and time of sacrifice determined by other blinded investigators. When moribund, mice were anesthetized with sodium pentobarbital, perfused intracardially with Trump's fixative (phosphate-buffered 4% formaldehyde/1% glutaraldehyde, pH 7.4), and spinal cords were removed and cut into 1-mm blocks. Blocks corresponding to mid-thoracic (T6) level were stained with osmium tetroxide, embedded in Araldite plastic (Polysciences, Warrington, PA) and 1-μm sections stained with 4% p-paraphenylene diamine to visualize myelin wrapping. An Olympus Provis AX70 microscope with a DP70 digital camera and a 60× oil-immersion objective was used to image six defined regions from each cross-section, sampling white matter from the anterior and lateral columns. All pathological analyses were performed without knowledge of the treatment group. Approximately 400,000 µm^2^ of white matter was sampled from each mouse. Myelin whorls, characteristic of degenerating axons, were counted in a blinded fashion from each SOD1G86R spinal cord from all sampled regions using ImageJ software (NIH). In addition, paraffin-embedded mid-thoracic and lumbar level tissue sections spaced by at least 1 mm were stained with an antibody against NeuN, a marker for neuronal cell bodies in the CNS. 5-µm sections were cut, de-paraffinized in xylene and rehydrated through a graded ethanol series to distilled water. Antigen retrieval and an avidin-biotin block (Vector Laboratories, Burlingame, CA) were performed before adding biotinylated anti-NeuN (Millipore, Temecula, CA). An avidin-biotin immunoperoxidase procedure detected the primary antibody (Vector Laboratories). The secondary antibody was revealed by a reaction mixture of DAB (3,3′-diaminobenzidine tetrahydrochloride) with hydrogen peroxide as a substrate (Sigma, St Louis, MO). Slides were dehydrated through a series of alcohol to xylene and cover slipped. Cells were counted from two thoracic and lumbar level sections from each mouse spinal cord.

### Neurological deficit performance assay

To assess the preservation of neurological function, five mice from each treatment group were randomly selected and monitored continuously as groups in Omnitech Accuscan locomotor activity-monitoring boxes (Omnitech Electronics, Columbus, OH). Nocturnal activity is a sensitive measure of spontaneous rodent neurological function. We previously reported that spontaneous nocturnal horizontal activity in groups of five mice provides a strong accurate measure of overall mobility, whereas nocturnal vertical activity provides a strong measure of rearing (Denic et al., 2011). This same publication described a preservation of deficits in the TMEV-induced model of spinal cord demyelination after a single dose of 200 μg of rHIgM12, which is how dosing for the ALS models was chosen. Mice were acclimated for 4 days prior to receiving a single 200 μg dose of rHIgM12 or control human IgM that does not bind to the surface of neurons at 60 days of age, and were then continuously monitored until the last mouse was moribund.

### Detection of rHIgM12 in the mouse brain and spinal cord

Isotopically labeled (^35^S) rHIgM12 was produced using Trans35S-Label (MP Biomedicals #51006) and validated for binding to the surface of primary cerebellar granule cell neurons in culture (Warrington et al., 2004). IgM was concentrated from culture supernatant by Polyethylene Gylcol and water precipitations, followed by re-suspension in normal saline and fractionation on a Superose-6 size exclusion column. Protein was quantitated by absorbance in a NanoDrop spectrophotometer and purity determined using denaturing and non-denaturing SDS PAGE. The acrylamide gel was exposed to autoradiography film to confirm radiolabel of light and heavy IgM chains. 1×10^7^ cpm (50 μg) of labeled IgM was administered intraperitoneally to groups of three 90-day-old SOD1G86R and age-matched normal FVB mice. Twenty hours later, plasma was harvested through the heart, mice were flushed transcardially with 50 ml of warm phosphate buffer and tissue acutely harvested. Tissues were weighed, a 50-100 mg portion dissolved in 0.5 ml of Solvable (PerkinElmer), incubated at 60°C for 4 h, 5 ml of Ultima Gold LSC liquid scintillation cocktail added per vial, mixed and cpm determined following the manufacturer's protocol. The ability of Solvable to lyse a given mass of tissue varies, so the manufacturer's guidance was used (LSC in Practice at Perkinelmer.com).

### Neuronal culture, immunocytochemistry and reagents

Cortical and cerebellar granule neurons from FVB mice were used. Pregnant mice were sacrificed under CO_2_ anesthesia and E15 embryos acutely removed for cortical cultures. Cerebellar granule cells were isolated from postnatal 5- to 7-day neonatal mice. Brain tissues were dissected at 4°C in HBSS buffer then digested with 0.1% trypsin at 37°C for 25 min. Tissue was then dissociated using a glass pipette in cold HBSS buffer. Neuronal density was calculated using a hemocytometer. Neurons were grown in neurobasal medium containing 2% (v/v) B27 supplement (Invitrogen, Carlsbad, CA) in a 5% CO_2_ incubator at 37°C. For neuraminidase treatment, neuraminidase from *Clostridium perfringens* (Neu-*C.P.*) was diluted in phosphate-buffered saline (PBS), pH 7.4 containing 0.1% BSA; cerebellar neurons were rinsed briefly with PBS and incubated with Neu-*C.P.* at 37°C for 4 h. For fluorescent immunocytochemistry, neurons were fixed with 4% paraformaldehyde in PBS, pH 7.4, and stained with primary antibodies followed by specific secondary antibodies or ligands. Fluorescence intensities were measured for each treatment and averaged within groups. Averages between groups were normally distributed. Antibodies used were: Alexa Fluor 594-cholera toxin B (C-34777, Life Technologies); alkaline-phosphatase-conjugated anti-human IgM (A2189, Sigma). Cortical neurons for biochemistry were seeded onto 35-mm tissue culture dishes coated with 4 µg/ml of laminin (Invitrogen) and 50 µg/ml of poly-D-lysine (Sigma, St Louis, MO).

### Western blot determination of tubulin modifications

Cortical neurons were used for western blot (Xu et al., 2011b). Briefly, neurons cultured in 35-mm dishes were treated with 10 µg/ml of MAG-Fc for 0, 30, 60 and 120 min. For combined treatment, 10 µg/ml of rHIgM12 and MAF-Fc mixture was added to media for 60 and 120 min. Treated neurons were then lysed simultaneously in ice-cold lysis buffer containing 50 mM Tris-HCl, pH 7.4, 150 mM NaCl, 1 mM EDTA, 1% Triton X-100 and protease-inhibitor cocktail (Roche Molecular Biochemicals, Indianapolis, IN). Neuronal lysates were mixed with sample buffer and denatured at 95°C. Neuronal lysates were separated on a 10% acrylamide gel, transferred to nitrocellulose and probed with anti-acetylated and anti-tyrosinated α-tubulin antibodies (Millipore Corporation, Bedford, MA). Immunoblots were analyzed by densitometry (Quantity One, Bio-Rad Labs, Hercules, CA) with protein levels normalized to α-tubulin. MAG-Fc was obtained from R&D Systems (Minneapolis, MN). Experiments were repeated three times.

### Thin-layer chromatography

For immuno-thin layer chromatography overlay assays (Saito et al., 2001), 20 μg of combined or single bovine-brain gangliosides (all from Sigma mixed gangliosides #G2250 80% gangliosides 11% N-acetyl neuraminic acid, GM1 #G9652>95%, GD1a #G2392>95%, GD1b #G8146>93%, GT1b #G3767>96%) were dissolved in chloroform/methanol (2:1) and separated on glass or aluminum-backed Silica gel 60 high performance thin-layer chromatography (HPTLC) plate (EMD Millipore, Darmstadt, Germany). Plates were fixed with poly-isobutylmethacrylate, cut into pieces and blocked with 0.1% bovine-serum albumin (BSA) in PBS, pH 7.4. Plates were first incubated with primary antibodies, washed and then incubated with alkaline-phosphatase-conjugated secondary antibodies. For neuraminidase treatment, HPTLC plates loaded with 20 μg of gangliosides were digested with 0.2 U/ml of neuraminidase (Worthington Biochemical, Lakewood, NJ) in PBS, pH 5.0 containing 0.1% BSA for 12-14 h. Treated plates were rinsed with PBS (pH 7.4) and overlaid with antibodies as above. Antibodies bound to the plate were visualized with NBT/BCIP reagents (Thermo Fisher Scientific). All antibodies were diluted in PBS, pH 7.4 containing 0.1% BSA. Binding studies were repeated at least three times.

### Nanohole surface plasmon resonance

For surface plasmon resonance (SPR)-binding experiments, we used custom chips and instruments (Im et al., 2011; Wittenberg et al., 2012). A thin metal film (100 nm Ag) with a large (8×8 mm) area square array of nanoholes (150 nm diameter, 500 nm periodicity) was formed by e-beam metal deposition onto a silicon mold with etched nanoholes, bonded to a glass slide with optical epoxy (NOA61) and template stripped off (Nagpal et al., 2009), forming the SPR sensor. A thin (∼15-25 nm) conformal hydrophilic SiO_2_ surface was deposited on the metal film by atomic layer deposition (Im et al., 2012) to enable bonding to an 8-channel flow cell made of polydimethylsiloxane (PDMS) (Sylgard 184, Dow Corning). Supported lipid bilayers (SLBs) with and without receptors were formed in the flow cells by the vesicle rupture method (Tamm and McConnell, 1985; Yang et al., 2001). Vesicles were made by mixing 1 mg lipids in organic solvents (chloroform/methanol, 2:1), evaporating the solvents (several hours under vacuum), and rehydrating in 0.5 ml aqueous buffer [100 mM NaCl, 10 mM tris(hydroxymethyl)aminomethane (Tris), 1 mM ethylenediaminetetraacetic acid (EDTA), pH 6.5]. Lipids used were L-α-phosphatidylcholine from chicken egg (egg PC, >99% pure, #840051C, Avanti Polar Lipids, Alabaster, AL), GD1a and GT1b from bovine brain (Sigma), GM1 from ovine brain (#860065, Avanti, >99% pure), and 1,2-dimyristoyl-sn-glycero-3-phosphoethanolamine-N-(lissamine rhodamine B sulfonyl) (RhoDMPE) (#810157, Avanti, >99%). SPR-experiment mixtures were egg PC (100 mol%), egg PC/GD1a (95/5 mol%), egg PC/GT1b (95/5 mol%) and egg PC/GM1 (95/5 mol%). For fluorescence recovery after photo bleaching (FRAP) experiments, we included 1 mol% RhoDMPE. Uniformly sized vesicles were made by vortexing, bath sonication (10 min) and extrusion (200 nm pore size, 15 passes, Avanti Mini-Extruder). The vesicles were then diluted to 1 mg/ml final lipid concentration by adding 0.5 ml Tris buffer with calcium (NaCl 100 mM, Tris 10 mM, CaCl_2_ 10 mM, pH 6.5), and the SiO_2_ surface was treated with oxygen plasma. Vesicles were applied to the surface and ruptured to form lipid bilayers, as confirmed by FRAP (Axelrod et al., 1976) experiments using an upright confocal microscope (Olympus FluoView 100 BX2). Vesicles in all studies were used within 8 days of preparation. During SPR experiments, all solutions (except for vesicles) were 0.01 M PBS, pH 6.5. SPR experiment analytes were rHIgM12 at 50, 100 or 200 nM or a control human IgM. The SPR sensor chip was incorporated into an instrument consisting of an inverted microscope (Eclipse Ti-S, Nikon), broadband light source, 2× objective (NA=0.06), imaging spectrometer (MS257, Newport Corp.) and CCD camera (PIXIS 400B, Princeton Instruments). Syringe pumps (PHD2000, Harvard Apparatus) injected solutions. Custom software collected and analyzed images: LabView (National Instruments), MATLAB (MathWorks) and OriginLab (OriginLab Corp.). An SPR-resonant dip in the optical transmission spectrum near 700 nm was fit with a fifth-order polynomial and the minimum was tracked over time to generate association and dissociation curves. SPR-binding experiments were carried out at room temperature (∼25°C). Binding-kinetic curve-fitting procedures were done as described previously (Kuziemko et al., 1996; Wittenberg et al., 2012). We fit a biphasic exponential decay curve to dissociation data, giving *k*_off,fast_ and *k*_off,slow_, then a monophasic exponential curve to association data to obtain *k*_on_ (using *k*_off,slow_), giving the apparent equilibrium dissociation constant, *K*_D,apparent_=*k*_off,slow_/*k*_on_.

### Statistical analysis

All numerical values shown are the mean±s.e.m. Mouse survival was plotted using Kaplan–Meier curves and statistical significance determined by Log-Rank test. Mann–Whitney Rank Sum test was used for data not normally distributed. Activity box performance data was exported and processed with Microsoft Excel and Prism (Graph-Pad Software Inc., San Diego, CA). The significance level was set as *P*<0.05 for all tests.

## Supplementary Material

Supplementary Material
