# Supplementary material for: A natural human IgM that binds to gangliosides is therapeutic in murine models of amyotrophic lateral sclerosis
Source: Dis Model Mech. 2015 Aug 1;8(8):831–42. doi: 10.1242/dmm.020727 (PMC4527295; doi:10.1242/dmm.020727)
Supplement: Supplementary Material [file supp_8_8_831__index.html]

Supplementary Material 

# A natural human IgM that binds to gangliosides is therapeutic in murine models of amyotrophic lateral sclerosis

## DMM020727 Supplementary Material

- Supplementary Material
