# Supplementary material for: A natural human IgM that binds to gangliosides is therapeutic in murine models of amyotrophic lateral sclerosis
Source: Dis Model Mech. 2015 Aug 1;8(8):831–42. doi: 10.1242/dmm.020727 (PMC4527295; doi:10.1242/dmm.020727)
Supplement: Supplementary Material [file supp_020727_DMM020727supp.pdf]

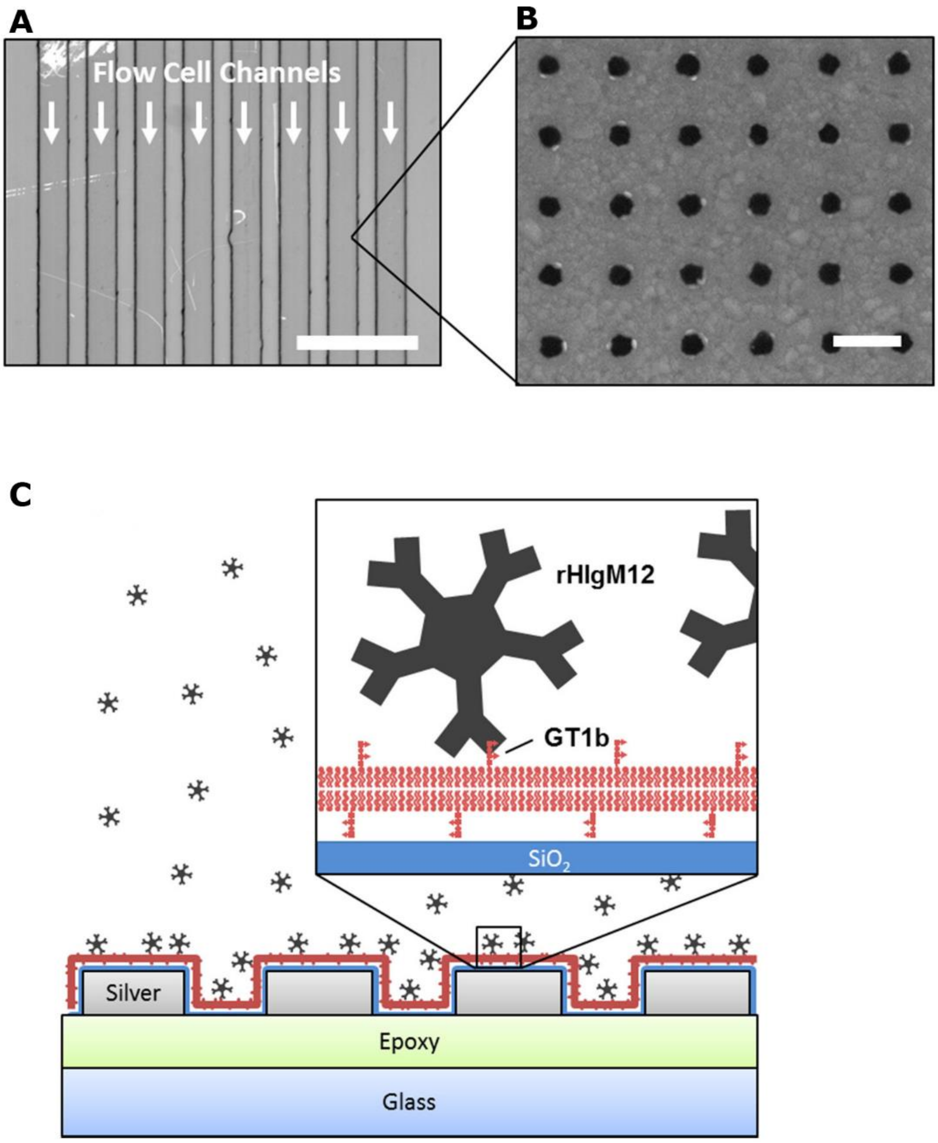

Suppl. Fig. 1. **Nanohole surface plasma resonance (SPR) technology designed to measure rHIgM12 binding to complex gangliosides**. A) Wide field optical micrograph of the PDMS microfluidic channels through which solutions are injected, over a large area (8×8 mm) nanohole array in a silver film. B) Scanning electron micrograph of the nanohole array. Nanoholes are 150 nm in diameter with 500 nm periodicity. C) Schematic cross section of the SPR sensor chip showing IgM binding to GT1b molecules in a SLB. An SiO_2 over layer provides a surface for lipid vesicles to rupture and form a planar lipid bilayer. rHIgM12 is injected over the surface and binds to GT1b and GD1a gangliosides. Broadband white light illuminates the nanohole array. Scale bar, (A) 500 µm; (B) 500 nm.

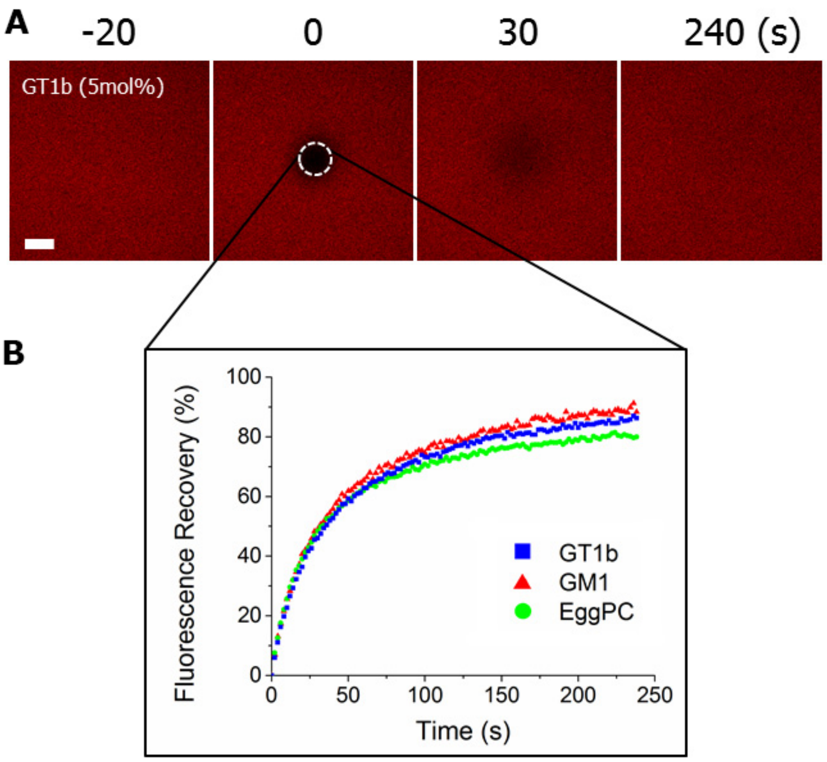

Suppl. Fig. 2. **FRAP verification of supported lipid bilayers** (SLB) formation on the SPR sensor. A) Fluorescent confocal microscope images over time of surface exposed to vesicles containing GT1b (5 mol%). B) Plot of fluorescence intensity in circular spot over time for multiple vesicle mixtures. Circular spot was bleached for 20 s. Recovery of intensity over time indicates vesicles ruptured to form a continuous bilayer. Vesicles were composed of egg PC with GT1b (5 mol %), GM1 (5 mol %), or no gangliosides. All mixtures contained Rho DMPE (1 mol %). Scale bar, 20 µm.

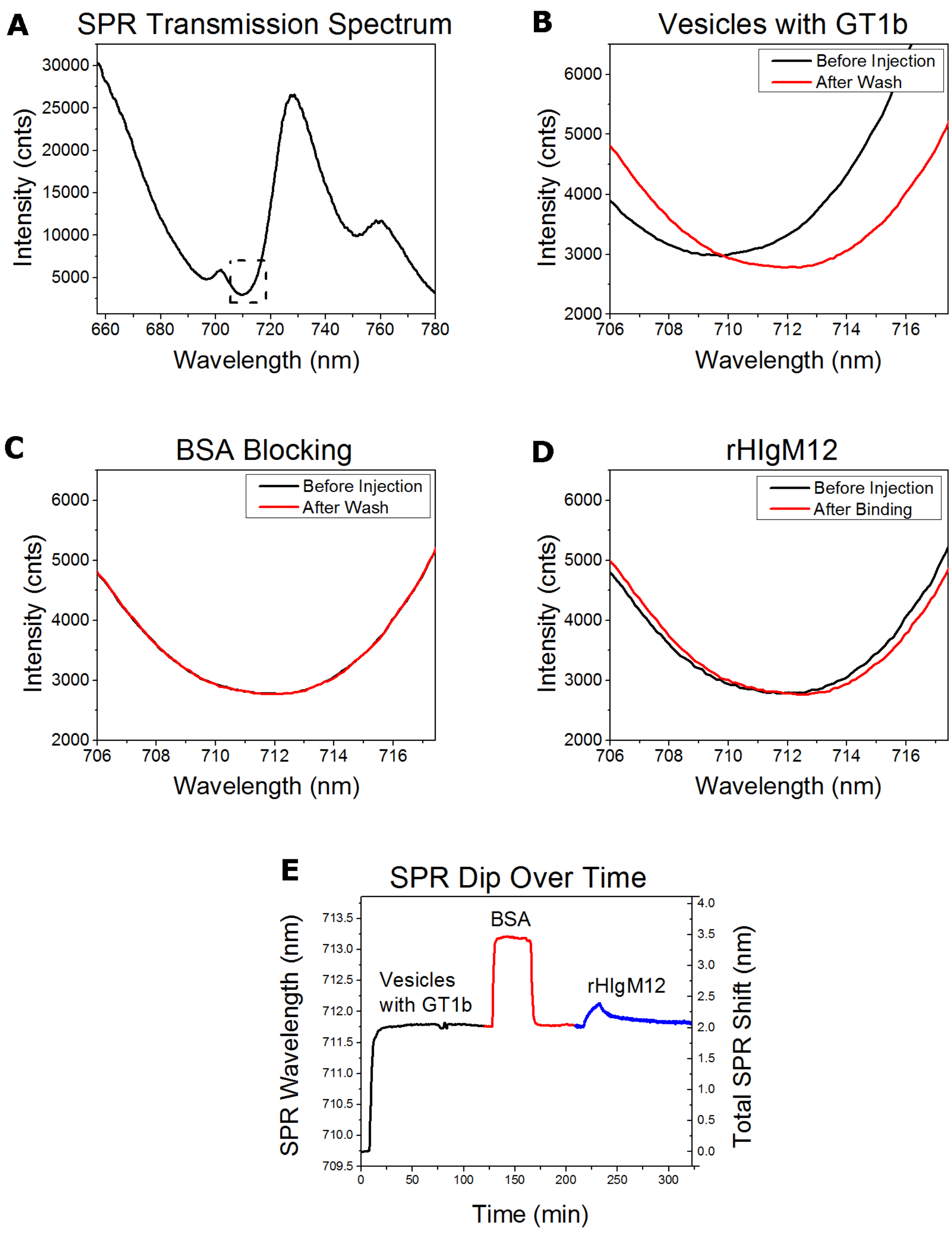

Suppl. Fig. 3. **SPR spectra of sample injections during SPR binding studies**. First, lipid vesicles were injected over the sensor and ruptured to form a lipid bilayer. **A**) The optical transmission spectrum of a nanohole array. The dashed box surrounds the SPR absorption feature tracked during (B-D). Close-ups show before and after reagent injections. **B**) Vesicles containing GT1b after and after wash step. **C**) Before and after BSA blocking step. **D**) Before

and after injection of rHIgM12. **E**) Plot of the central wavelength of the SPR absorption feature in time for each step. Association between molecules was detected by the shift of the SPR absorption during analyte injection. PBS was used to dissociate the complex, resulting in a reversal of the shift. Note that the sensor response during BSA injection is large due to the high concentration (20 mg/mL) and that a negligible amount of BSA absorbs during the blocking step (as shown by the recovery of the initial condition) indicating high lipid-bilayer coverage.
